# Searching QTL by gene expression: analysis of diabesity

**DOI:** 10.1186/1471-2156-6-12

**Published:** 2005-03-10

**Authors:** Aaron C Brown, William I Olver, Charles J Donnelly, Marjorie E May, Jürgen K Naggert, Daniel J Shaffer, Derry C Roopenian

**Affiliations:** 1 The Jackson Laboratory, 600 Main Street, Bar Harbor, Maine 04609, USA

## Abstract

**Background:**

Recent developments in sequence databases provide the opportunity to relate the expression pattern of genes to their genomic position, thus creating a transcriptome map. Quantitative trait loci (QTL) are phenotypically-defined chromosomal regions that contribute to allelically variant biological traits, and by overlaying QTL on the transcriptome, the search for candidate genes becomes extremely focused.

**Results:**

We used our novel data mining tool, ExQuest, to select genes within known diabesity QTL showing enriched expression in primary diabesity affected tissues. We then quantified transcripts in adipose, pancreas, and liver tissue from Tally Ho mice, a multigenic model for Type II diabetes (T2D), and from diabesity-resistant C57BL/6J controls. Analysis of the resulting quantitative PCR data using the Global Pattern Recognition analytical algorithm identified a number of genes whose expression is altered, and thus are novel candidates for diabesity QTL and/or pathways associated with diabesity.

**Conclusion:**

Transcription-based data mining of genes in QTL-limited intervals followed by efficient quantitative PCR methods is an effective strategy for identifying genes that may contribute to complex pathophysiological processes.

## Background

Understanding the molecular etiology of disease processes is a pressing goal of 21^st ^century medicine. Completion of the mouse genome holds considerable promise in the discovery of genes responsible for genetically determined complex diseases. Quantitative trait loci (QTL) are allelically variant regions detected by virtue of their contribution to the overall complex disease phenotype and thus are "experiments in nature", which mark chromosomal intervals carrying genes with a proven disease involvement. Since gene expression is a key link between the genome and the plethora of phenotypic traits exhibited, tools that permit the analysis of the tissue expression pattern of genes in their chromosomal context provides a bridge between QTL and the genes responsible.

Global microarray gene expression technologies offer a promising, unbiased approach toward this goal in that they reveal gene expression changes, which can be correlated with the disease phenotype. However, such global methods of analysis are not routine analytical tools and can suffer from incomplete gene coverage, as well as lack of sensitivity. Because only a small fraction of the transcriptome is typically involved in any given etiopathological process, bioinformatic data mining tools that allow for the intelligent prioritization of genes would make it possible to employ more routine and sensitive expression technologies, such as quantitative polymerase chain reaction (QPCR). ExQuest [1,2] organizes pre-existing information-rich expression databases in a way that quantitative tissue and/or developmental gene expression patterns can be extracted and displayed within the context of whole chromosomes. By overlaying ExQuest "chromosomal expression maps" with QTL coordinates, one can search within defined genomic intervals for candidate genes with enhanced expression in tissues consistent with disease pathology. Screening candidates by QPCR will determine whether or not they exhibit expression changes in genetically resistant versus susceptible mice.

Type II diabetes (T2D), also referred to as non-insulin dependent diabetes mellitus, is characterized by insulin resistance of target cells combined with insufficient insulin production and/or abnormal secretion by pancreatic β-cells, which eventually results in chronically elevated glucose levels in the circulation. Obesity is a major risk factor for T2D, and the term "diabesity" has been coined to collectively describe these overlapping conditions [3]. This paper describes an ExQuest compilation of novel candidate genes from mouse diabesity QTL intervals mapped to chromosomes 1, 4, 10, 17, 19 and X. This is followed by QPCR analysis of the ExQuest candidates, in addition to known diabetes and metabolism genes selected from the literature, using tissues derived from the newly defined T2D model, Tally Ho (TH) [4]. By selecting genes from QTL intervals derived from multiple diabesity models and using the TH mouse as a susceptible strain to test for differential expression, we have identified a number of genes whose altered expression may be involved in the development of diabesity.

## Results

### Diabesity-relevant candidate gene selection

**Figure 1:**
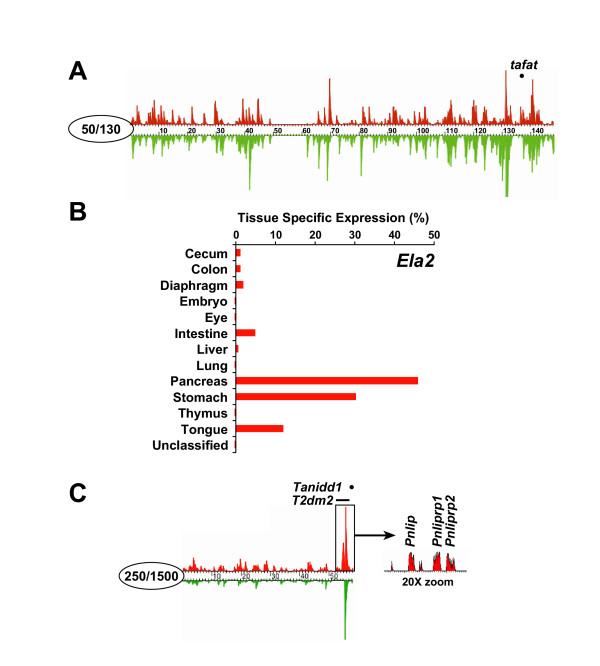
Mirrored tissue-specific expression (red) and absolute EST representation (green) for whole ExQuest chromosomes. Maximum scales set for specific or absolute expression is within the circle at the centromere. (a) Pancreas expression for mouse chromosome 10 showing peak position of the LOD for the QTL *Tafat *over a pancreas expression peak that contains the gene *Ela2*. (b) Quantitative tissue specific expression profile for the gene *Ela2*. Results are derived from the number of *Ela2*-aligned EST hits normalized for library density. (c) Pancreas expression for mouse chromosome 19. Genes in the *Tinidd1 *and *T2dm2 *region are pancreas specific (red) and highly expressed (green). Arrow shows a 20X zoom of 3 individual genes *Pnlip *(1810018F18Rik), *Pnliprp1 *and *Pnliprp2*, which contribute to the large pancreatic histogram peak.

To identify candidate genes that contribute to diabesity, we first established the local boundaries of 29 QTL from the LocusLink database that contribute to body weight, adiposity or T2D, and map to mouse chromosomes 1, 4, 10,17, 19 and X (Table 1). Using ExQuest, we were able to cluster publicly available ESTs to genomic fragments, extract and normalize tissue information from EST datasheet records, and display quantitative expression information linearly along the six chromosomes for over 70 tissues. We then narrowed the chromosomal search by overlaying the *in silico *expression data with QTL intervals. We were particularly interested in genes with known or potential metabolic function whose expression patterns were biased towards high expression in three diabesity relevant-tissues, pancreas, liver and adipose tissue, for which there was good EST library representation. (Skeletal muscle was not included due to the lack of available mouse EST libraries.) An example is illustrated in Figure 1A, in which a region of genes with expression strongly biased toward pancreatic tissue is found centered over the 1-LOD 95% confidence interval of the TH QTL *Tafat *[4]. By using ExQuest's zoom-in capability, resolution down to the expression of individual exons revealed that the expression bias was explained by a single gene, Elastase 2 (*Ela2*), with a pattern of expression that includes pancreas, stomach and tongue (Fig. 1B). A second example is illustrated in Figure 1C, in which a cluster of pancreatic expressing genes is localized to two chromosome 19 T2D QTL, *T2dm2 *[5] and *Tanidd1 *[4]. The expanded chromosomal view revealed that this cluster contained pancreatic lipase (*Pnlip*) and two paralogs, *Pnliprp1 *and *Pnliprp2*. From all QTL intervals, we chose the 71 "best expression" candidates (Table 1) out of a possible 4,013 genes as predicted by Ensembl's gene annotation. From PubMed searches, we selected an additional 24 genes considered to be highly relevant to diabesity and/or metabolism, as well as the standard normalizer 18S RNA, bringing our total to 96, a convenient number for QPCR profiling (for gene list and oligonucleotide primer sequences, see Additional file 1).

**Table 1:** Genes selected for expression analysis by ExQuest.

**Chr**	**QTL**^1^	**Genes picked via ExQuest**	**Total Genes in Region**	**Interval Range (Mb)**
1	Obq2	Gsta3	73	13.7
	*Obq7*, *Wt6q1*, *Insq2*, *Insq6*	*Aox1*, *Fn1*, *Pecr*, *Igfbp2*, *Plcd4*, *Scg2*, *IRS-1*, *Inpp5d*	401	44.1
	*Nidd6*	*Qscn6*	85	9.4
	*Obq9*	*Fmo1*, *Fmo3*, *Apoa2*	117	11.7
	*Wt6q2*	*Hsd11b1*	63	7.3
4	*Bwq1*	*Decr1*	64	12.1
	*Triglq1*, *Bglq4*	*Ttpa*, *Bhmt*, *Baat*, *Aldo2*	40	9.8
	*Nidd1*, *Dob1*	*Lepr*, *Dio1*, *Scp2*, *Faah*, *Usp1*	350	32.4
	*Tafat*	*Ela3b*, *Ela2*, *Dvl1*	319	18.0
10	*Insq9*, *Igfbp3q2*	*Ass1*, *Ftcd*, *Col18a1*, *Itgb2*, *Agpat3*, *Ndufs7*, *Oaz1*, *Pah*, *Igf-1*, *Nr1h4*, *Kitl*	605	55.1
	*Bgeq8*	*Rdh7*, *Hsd17b9*	257	19.8
17	*Obq4*, *Wta4*	*Plg*, *Acat2*, *Actl*, *Hagh*, *Igfals*, *Decr2*, *Clps*, *Tff1*, *Tff2*, *Tff3*, *Apom*	554	27.2
	*Hdl4*	*Gnmt*, *Lrg*, *Sepr*	161	10.7
	*Insq5*	*Abcg5*	69	10.0
19	*Iba4*	*Aldh1a1*, *Aldh1a7*, *Vldlr*, *Plce1*	190	23.3
	*Afw8*	*Pi4k2a*, *Cpn1*, *Elovl3*	159	8.8
	*Nobq2*, *Bglq13*	*Ins1*, *Gpam*, *Facl5*	39	8.4
	*Tanidd1*, *T2dm2*	*Gfra1*, *Pnlip*, *Pnliprp1*, *Pnliprp2*	40	3.6
X	*Bw1*	*Rgn*	149	51.5
	*Bw3*	*Rab9*	278	30.0

^1^QTL name, abbreviation: Obesity, *Obq*; Body weight, 6 weeks, *Wt6q*; Insulin, *Insq*; Non-insulin-dependent diabetes mellitus, *Nidd*; Body weight, *Bwq*; Triglyceride, *Triglq*; Body growth late, *Blgq*; Dietary obesity, *Dob*; Tally ho associated mesenteric fat pad weight, *Tafat*; Insulin-like growth factor binding protein 3, *Igfbp3q*; Body growth early, *Bgeq*; Weight adult, *Wta*; High density lipoprotein level, *Hdl*; Induction of brown adipocytes, *Iba*; Abdominal fat weight, *Afw*; New Zealand obese, *Nobq*; Tally ho associated non-insulin dependent diabetes mellitus, *Tannid*; Type 2 diabetes mellitus, *T2dm*; Body weight, *Bw*.

### Real-time QPCR profiling

The newly-described TH T2D mouse model develops obesity, hyperinsulinemia, hyperlipidemia, and male-limited hyperglycemia [4]. To test the validity of our candidate gene selection and determine a quantitative molecular signature for this mouse model, we extracted adipose, liver and pancreas RNA from three early diabetic (as verified by glycemic index) 7-week-old male TH mice, as well as three age and sex matched B6 controls. Real-time QPCR was then independently performed for all 96 genes on the three biological replicates, and the results were processed using GPR software (for raw Ct values and GPR Reports, see Additional files 2, 3 and 4). Twenty-one of 96 genes exhibited significant expression changes between TH and control B6 mouse tissues (Table 2). Fifteen were among the 71 candidates chosen by ExQuest, while six were from the 24-gene PubMed pool (Table 3).

**Table 2:** GPR report of B6 vs. TH gene expression comparisons.^1^

**Gene**	**# Total hits^2^**	**GPR Score^3^**	**Fold change^4^**
	**Adipose**		
Pparg	44	0.92	-14.9
*Lep*	40	0.83	5.3
*Alb1*	39	0.81	-9.4
*Gsta3*	38	0.79	-10.6
*Glut2*	37	0.77	-28.1
*Fmo1*	35	0.73	-4.8
*Scp2*	25	0.52	-2.4
*Agpat2*	24	0.50	-2.9
*Agpat3*	19	0.40	-2.1
			
	**Pancreas**		
*Ela2*	39	0.95	-55.8
*Pnliprp2*	39	0.95	-21.8
*Kitl*	29	0.71	-4.3
*Col18a1*	18	0.44	-1.7
*Ela3b*	16	0.40	-1.8
			
	**Liver**		
*Tff3*	63	1.00	-593.0
*Vldlr*	58	0.92	15.6
*Hsd17b9*	57	0.91	9.5
*Pparg*	56	0.89	-11.5
*Igfbp2*	46	0.73	-2.7
*Kitl*	39	0.62	-2.4
*Decr2*	28	0.44	-1.3
*Lpl*	25	0.40	-1.2
*Pi4k2a*	25	0.40	-1.1

^1 ^Complete GPR report and raw CT data in Supplementary Data.

^2^Number of qualified normalizer genes against which test gene ΔC_T _values are statistically significant at p = 0.05.

^3 ^# of total hits / # of qualified normalizers. A GPR score = 0.40, meaning that the test gene ΔC_T _comparisons differed at p= 0.05 compared with at least 40% of the qualified normalizers, is considered most reliable.

^4^Relative to 18S RNA.

**Table 3:** Medline citations to genes showing altered expression in TH mice.

**Gene name**	**Diabesity-relevant articles**	**General function**
Genes chosen known to be involved in diabetes with expression change		
Alb1	5548	Lipid binding and carrier activity
*Leptin*	3170	Hormone activity
*Lpl*	775	Lipid catabolism or fatty acid metabolism
*Pparg*	702	Lipid metabolism and steroid hormone receptor activity
*Glut2*	142	Glucose transport and carbohydrate metabolism
*Agpat2*	5	Phospholipid biosynthesis
		
ExQuest chosen genes with expression change, known or unknown to be involved in diabetes		
*Col18a1*^1^	7	Potent antiangiogenic protein, structural molecule
*Vldlr*^2^	7	Lipid transport
*Scp2*	4	Sterol carrier activity, lipid binding, and steroid biosynthesis
*Fmo1*	3	Electron transport and oxidoreductase activity
*Pnliprp2*	1	Lipid catabolism
*KitL*	0	Signal transduction
*Igfbp2*	0	Insulin-like growth factor binding
*Ela2*	0	Proteolysis and peptidolysis
*Ela3b*	0	Proteolysis and peptidolysis, and cholesterol metabolism
*Gsta3*	0	Biosynthesis of steroid hormones
*Agpat3*	0	Phospholipid metabolism
*Hsd17b9*	0	Steriod biosynthesis and oxidoreductase activity
*Decr2*	0	Peroxisome organization and biogenesis, and oxidoreductase activity
*Tff3*	0	Epithelium healing
*Pi4k2a*	0	Inositol/phosphatidlinositol kinase activity
		
**Functional summary of genes with expression changes**^3^		
Lipid related gene activity	8	
Hormone related gene activity	6	
Inflammation or response to injury	4	
Oxidoreductase activity	3	
Signal transduction	3	
Proteolysis and peptidolysis	2	
Carbohydrate related gene activity	1	

^1^*Hsd17b9 *medline search: there are 88 articles for the homolog "11-beta hydroxysteroid dehydrogenase", and 157 with "hydroxysteroid dehydrogenase".

^2 ^*Vldlr *MEDLINE search: 4818 diabesity relevant articles found for *Vldl*.

^3 ^Genes with dual functions are counted twice.

### Novel genes potentially implicated in the progression or response to diabetes

To assess the novelty of the differentially expressed ExQuest-selected genes in regard to diabesity, we performed an extensive PubMed search for articles associated with both the gene name (or its alternative nomenclature as defined by LocusLink gene name aliases), and diabetes or obesity. The number of articles retrieved were typically one to two orders of magnitude lower for the differentially expressed ExQuest selected genes in comparison to the genes analyzed with known diabesity or metabolism involvement, with many having no literature citations (Table 3). QTL-based ExQuest expression mining thus revealed a number of novel diabesity candidate genes with little to no prior association with this disease.

## Discussion

Our results suggest that *in silico *data mining focused on gene expression in diabesity-affected tissues and limited to intervals containing diabesity-specific QTL (and thus enriched in genes that contribute to diabesity) is an efficient method to identify genes whose expression is altered in T2D-susceptible mice. This analysis not only demonstrated expression alterations in genes known to be associated with the development of diabesity, but also identified a number of novel genes whose expression changes may contribute to the development of diabesity. Expression changes in genes mapping within TH QTL could be considered as candidates for transcriptional polymorphisms contributing to the associated TH QTL. However, genes selected on the basis of other diabesity QTL that showed transcriptional differences in the TH/B6 comparison are more likely minor QTL in the TH model or symptomatic effects of diabesity rather than a major genetic cause of the TH disease.

As other diabesity studies commonly observe, there were highly significant changes in adipose tissue expression of *Pparg *and *Lep*. This included a ~5-fold increase in leptin expression in TH adipose with no accompanying increase in expression of the leptin receptor (*Lepr*). Since leptin is responsible for the regulation of food intake [6], this increase is most likely in response to incipient weight gain. In contrast, the observed decrease in the expression of peroxisome proliferator activated receptor gamma (*Pparg*), both in TH adipose tissue and liver, is consistent with a decrease in adipocyte differentiation often observed in obese states [7,8]. In addition, *Pparg *agonists are generally associated with promoting insulin sensitization in the context of obesity [9].

Other genes previously associated with diabesity and showing differential expression in TH vs. B6 tissues included a 28.1-fold decrease of glucose transporter 2 (*Glut2*) in adipose, a 2.9-fold decrease in 1-acylglycerol-3-phosphate O-acyltransferase 2 (*Agpat2*) in adipose, and a 1.2-fold decrease in lipoprotein lipase (*Lpl*) in liver. Their reduced expression in the TH model might be in response to disease onset.

Serum albumin levels are decreased in T2D patients (reviewed in [10]). The primary source of serum albumin is liver. Albumin synthesis is decreased in both diabetic humans and in rat T2D models[11,12] and in liver cells deprived of insulin [13]. We failed to detect any alteration in the normally high levels of *Alb1 *expression in liver, but TH mice showed a 9.4-fold decrease in *Alb1 *expression in adipose tissue. Whether this fat-specific decrease in *Alb1 *expression is an early manifestation of subsequent hypoalbuminemia remains to be established.

Altered expression of a number of ExQuest-selected genes was also found. TH livers showed a 15.6-fold expression increase compared with B6 for the very low-density lipoprotein receptor (*Vldlr*). As a deficiency in *Vldlr *has been reported to reduce adipocyte size and obesity in the *ob/ob *mouse model [14], this expression alteration may contribute to TH diabesity. The expression of hydroxysteroid (17β) dehydrogenase 9 (*Hsd17b9*), which localizes to the early body weight QTL *Bgeq8 *QTL, was elevated 9.5-fold in TH livers. While the function of this gene is not described in the literature, its paralog 11beta-hydroxysteroid dehydrogenase type 1 (*Hsd11b1*) bidirectionally catalyzes the conversion of cortisol to the inactive metabolite cortisone. Over 150 articles describe an association of *Hsd11β1 *with diabesity, and an intronic *Hsd11b1 *polymorphism is associated with obesity and insulin resistance in children [15]. However, whether the elevated expression of the *Hsd17b9 *paralog in TH liver is a candidate for *Bgeq8 *remains to be established.

Exocrine secretion of pancreatic lipases are known to hydrolyze triglycerides to free fatty acids in the small intestine. Pancreatic lipase (*Pnlip*) and two paralogs, *Pnliprp1 *and *Pnliprp2*, were expressed preferentially in pancreas and mapped very close to the peak of the LOD values for the overlapping diabesity QTL, *T2dm2 *and *Tannidd1 *(Fig. 1b). *T2dm2 *and *Tannidd1 *are responsible for increased insulin levels [5] and elevated plasma glucose [4], respectively. Long-term high fat feeding, leading to glucose intolerance, occurs with a simultaneous decrease in mRNA expression of *Pnlip *[16]. While no expression alteration in the normally high levels of expression of *Pnlip *or its closely related *Pnliprp1 *paralog were found, *Pnliprp2 *expression was decreased 21.8-fold in TH pancreas when mice were fed a 4% fat diet. By facilitating fat storage, the consequence of which is hyperglycemic diabetes, the selective decrease in *Pnliprp2 *in TH mice may explain *Tanidd1*, and potentially, the genetically overlapping *T2dm2 *QTL.

*Trefoil 3 (Tff3*), which maps to the *Obq4 *obesity QTL, was the most significantly changed of all genes analyzed. It is quite transcriptionally active in control B6 liver but virtually undetectable in TH liver. Trefoils are small, stable secretory proteins expressed in goblet cells in the gastrointestinal mucosa where they stabilize the mucus layer and promote epithelial healing [17]. Mice deficient in *Tff3 *are highly susceptible to colon damage [18], which is not commonly associated with T2D. While the colon is the primary tissue of expression, *Tff3 *has also been reported to be expressed in the bile ducts of normal human liver and is upregulated in diseased livers [19]. However, a potential association of the remarkable reduction of *Tff3 *expression in TH liver with diabesity remains to be established.

While not detectably expressed in the liver or fat tissue in B6 or TH mice, *Ela2 *was ranked by GPR as the gene most significantly changed in the pancreas, with a 55-fold reduction in TH vs. B6 mRNA (Table 2). Originally cloned from the pancreas [20], *Ela2 *is located within 75 kb of the peak LOD score for the TH QTL *Tafat*. ExQuest expression profiling suggests that *Ela2 *expression is biased strongly towards digestive tissues (pancreas, stomach and tongue) of normal mice (Fig. 1A). Both whole pancreas and Islet of Langerhans EST libraries show high levels of *Ela2 *expression, suggesting that the endocrine pancreas actively transcribes it. *Ela2 *(alias polymorphonuclear neutrophil elastase) encodes a serine protease present in neutrophil endosomal granules, which are known to be important in myelopoiesis (reviewed in [21]). The substantially decreased expression in TH pancreas may result in secretory granules deficient in this serine protease.

## Conclusion

We have tested the concept that transcription-based ExQuest data mining of genes in QTL-limited intervals is an effective method to identify genes that contribute to the complex genetic disease, diabesity. By limiting the *in silico *candidate genes to those with expression biased to tissues normally affected by this disease, we have shown that sensitive, high-throughput QPCR methods reveal expression changes in novel genes in the TH model. The search within additional diabesity QTL using this technique may facilitate the identification of a limited number of genes that comprise a 'complete' diabesity molecular phenotype. Moreover, this general approach may be an efficient method to identify genes that contribute to complex pathophysiological processes.

## Methods

### QTL analysis

Obesity and T2D QTL on mouse chromosomes 1,4,10,17,19, and X, were identified by a LocusLink search [22]. Information regarding individual QTL boundaries was gathered through the Mouse Genome Informatics site (MGI) [23]. Simple sequence length polymorphism (SSLP) markers extracted from MGI were queried at the Ensembl website [24] to determine the exact chromosomal location.

### In silico expression analysis

ExQuest [1,2] is a gene expression program that uses public EST databases to create a comprehensive transcriptome map overlaid with tissue specific expression. In brief, chromosomal sequence was downloaded from Ensembl and stringently masked using the RepeatMasker program. ESTs were clustered to the chromosomes using the MegaBlast algorithm on a 32-node, 64 cpu Beowulf cluster. Tissue or library source information was extracted from EST datasheet records for each 10,000 base pair genomic fragment and normalized based upon library density and EST hit frequency. Whole chromosomal plots display total EST hits for each tissue as well as normalized data, which is a measure of a specific tissue expression level compared to all other tissues available in dbEST. In this way, the expressional bias of a genomic region towards a specific tissue is determined. QTL intervals were overlaid onto the chromosomal plots displaying adipose, liver and pancreas expression and these regions were then scanned for areas that exhibited high tissue specificity. ESTs clustering to genomic regions showing high tissue specificity were linked to Unigene [25], to determine if the particular EST aligned to a known gene cluster from which mRNA sequence could be extracted for primer design.

### Tissue procurement, RNA preparation and cDNA synthesis

Three 7 week old, male TH mice (nonfasting blood glucose levels 295 to 433 mg/dl) and their respective T2D-resistant, sex- and age-matched C57BL/6J (B6) controls, were maintained on a 4% fat diet [4]. Following CO_2 _asphyxiation, pancreas, adipose, and liver tissues were collected. Each biological replicate sample was processed in parallel. Whole pancreas tissue was collected, placed in 3.5 mL lysis/binding solution from the RNAqueous^®^-4PCR Kit (Ambion # 1914), homogenized and stored in an ethanol/dry ice bath until the RNA could be extracted. Approximately 25 mg of adipose and liver tissue were collected and stored in 1 mL of RNALater (Ambion # 7020) until RNA was extracted.

For pancreatic RNA extraction, we prepared a 5.7 M CsCl solution containing 0.05 M EDTA, pH 7.0. The solution was made RNase free using RNASecure (Ambion # 7005), conforming to manufacturer's recommendations. 5.0 mL SW 55.1 ultracentrifuge tubes were made RNase free by treatment with RNAZap (Ambion # 9780) for five minutes followed by washing with Nuclease-Free water (Ambion # 9932). 2.0 mL of the 5.7 M CsCl solution was added to each tube. 3.0 mL of pancreatic solution was layered carefully on top. The tubes were centrifuged at 36,000RPM for 16–20 hours at 25°C in a SW 55.1 rotor. The supernatant was carefully removed 1.0 mL at a time, using fresh pipette tips. When approximately 1.0 mL remained, the tubes were quickly inverted and dried with filter paper to prevent residual RNases from contact with the RNA pellet at the bottom of the tube. The RNA was resuspended in 2 portions of 150 μL of RNA resuspension solution (Ambion #7010), conforming to manufacturer's recommendations.

Adipose and liver tissues stored in RNAlater were processed using the RNAqueous^®^-4PCR Kit, conforming to the manufacturer's recommendations.

All RNA was subsequently digested with DNAse in accordance with the aforementioned protocol and analyzed for purity using the Agilent Bioanalyzer 2100. RNA concentrations were determined using a NanoDrop^® ^ND-1000 Spectrophotometer. The collected RNA was converted into cDNA via MessageSensor™ RT Kit (Ambion # 1745), conforming to manufacturer's recommendations. The adipose tissue cDNA reaction synthesis contained 350 ng of RNA while pancreas and liver samples utilized 1000 ng of RNA. All RNA was stored at -80°C when it was not being processed.

### Primer design

Sequences for the candidate genes were extracted from GenBank and imported into Primer Express software v2.0 from Applied Biosystems, Inc (ABI). All primers were prepared in accordance with universal thermocycling parameters as described for real-time PCR on the ABI 7900HT. The primer sequences were then blasted [26] to ensure specificity of the primers. Forward and reverse primers (MWG Biotech) were combined in a 96-well master plate at a final concentration of 50 μM. Short amplicons of approximately 75 bp were generated to ensure a high level of sensitivity. Dissociation curves confirmed that only single amplicons were generated. Amplicons were TA cloned (Invitrogen # K204040) and bidirectionally sequenced to confirm sequence identity for instances in which genes exhibited significant expression. For gene names and primer sequences, see Additional file 1.

### Real-Time QPCR

Each reaction consisted of 5.0 μl of 2X SYBR Green Master Mix (ABI # 4309155), 3.0 μL of dH_2_O, 1.5 μL of 0.5 μM forward and reverse primer solution, and 0.5 μL of cDNA. Pancreas and liver cDNA was diluted 1:10 before addition to the master-mix, while adipose cDNA was diluted 1:3. A 384-well plate format was utilized such that 4 samples × 96 genes were amplified per plate. The plate was sealed with Optical Adhesive Covers (ABI # 4311971) and centrifuged. The samples were assayed on the ABI Prism 7900HT Signal Detection System v2.0 using default conditions, and baseline range values were set from 3 to 10 cycles.

The data were then analyzed using Global Pattern Recognition (GPR) analytical software [27,28]. In typical QPCR experiments, the comparative expression of all genes is based on single gene normalizer, whose expression is assumed to be invariant. In contrast, the Global Pattern Recognition (GPR) algorithm employs a global normalization feature in which the expression data from each gene are normalized against that of every other gene, thus eliminating the reliance on single gene normalization. GPR's ranking is based on biological replicate consistency, and is thus not skewed by fold change in the magnitude of expression. For raw CT values and GPR Reports, see Additional files 2, 3 and 4.

## Abbreviations used in this paper

QPCR, quantitative polymerase chain reaction; QTL, quantitative trait loci; GPR, global pattern recognition; ExQuest, expressional quantification of ESTs

## Authors' contributions

ACB and DCR conceived of the study and were primarily responsible for its coordination and design. ACB and WIO were responsible for maintenance and execution of software algorithms as well as candidate gene selection. WIO and DJS preformed all tissue extraction and QPCR. CJD and MEM provided supercomputer and database management. JKN provided all mice, animal husbandry, and diabesity technical expertise. ACB, WIO and DCR drafted the manuscript and figures.

## Supplementary Material

Additional File 1 Gene names and primer sequences used for QPCR. Click here for file

Additional File 2 Provides adipose tissue raw Ct values and GPR report. Click here for file

Additional File 3 Provides liver raw Ct values and GPR report. Click here for file

Additional File 4 Provides pancreas raw Ct values and GPR report. Click here for file
